# German translation and validation of the complementary and alternative medicine health belief questionnaire (CHBQ)

**DOI:** 10.1186/s12909-023-04985-9

**Published:** 2024-01-30

**Authors:** Maximilian Hinse, Lea Jerzynski, Sylvia Binting, Sonja Kummer, Benno Brinkhaus, Gabriele Rotter

**Affiliations:** 1 Institute of Social Medicine, Epidemiology and Health Economics, Charité – Universitätsmedizin Berlin, Corporate Member of Freie Universität Berlin and Humboldt-Universität zu Berlin, Charitéplatz 1, 10117 Berlin, Germany; 2 Department of Pediatric Oncology and Hematology, Charité – Universitätsmedizin Berlin, Corporate Member of Freie Universität Berlin and Humboldt-Universität zu Berlin, Augustenburger Platz 1, 13353 Berlin, Germany

## Abstract

**Background:**

The Complementary and Alternative Health Belief Questionnaire (CHBQ) measures medical students’ attitudes towards Complementary Medicine (CM). The aim of the study was to examine the validity and reliability of the German translation of the CHBQ.

**Methods:**

Data for the psychometric evaluation of the German translation were drawn from a study that investigated attitudes towards CM in (a sample of) medical students at Charité - Universitätsmedizin Berlin. Construct validity was determined via an exploratory factor analysis (EFA). Internal consistency was examined using Cronbach’s alpha and split-half reliability.

**Results:**

The CHBQ was returned by 278 students, and was fully completed by 260 students (mean age 23.7 years; ± 4.3 SD), 69.2% were female). EFA revealed a single factor solution for all 10 items of the scale. All items, except one, had good item discrimination (range: 0.5–0.8), acceptable mean inter-item-correlation (0.39) and similar median correlation (0.38). Reliability was very good (α = 0.86) and further confirmed by split-half reliability (0.91).

**Conclusions:**

The German version of the CHBQ is a valid and reliable instrument for measuring students’ attitudes towards CM.

## Introduction

Complementary Medicine (CM), a definition that until a few years ago was partly used synonymously with Complementary and Alternative Medicine (CAM), covers a heterogeneous group of diagnostic and therapeutic procedures [1] for which there is at least of some evidence of efficacy. Nevertheless, for approximately 10 years a the newer term has been used, namely: Complementary and Integrative Medicine or Complementary and Integrative Heath. In the United States of America (USA), this is exemplified by the National Center for Complementary and Integrative Health (NCCIH, [2]). Other terms such as naturopathy or natural medicine are also used [3–6]. CM includes, for example, acupuncture, manual therapies such as osteopathy and herbal remedies. CM can be combined with conventional medicine.

CM has been found to be widely used by patients. The prevalence of CM utilization ranges from 9.8 to 76% [7]. In Germany, CM is offered by more than 60–80% of physicians [8, 9] and it is increasingly integrated into the undergraduate medical education curriculum. However, there is an international lack of objective and reliable instruments to educate medical students in CM [10].

In the United States, questionnaires specifically designed for medical students have been developed and validated to assess students’ and health professionals’ attitudes towards CM [11, 12]. In 2003, the 29-Item Integrative Medicine Attitude Questionnaire (IMAQ) was validated in English. The shorter 10-item CAM Health Belief Questionnaire (CHBQ) was developed by Lie and Boker [11] and validated in medical students in the USA. The CHBQ was found to be a practical, valid, and reliable instrument (alpha = 0.75) for measuring medical students’ attitudes and health beliefs. It was found to be potentially useful for measuring the impact of CM education [13]. Since then, the CHBQ has been used internationally, also in non-medical students [11, 12, 14–17]. To the best of our knowledge and based on a literature search, our research group was the first in Germany to use a translated German version of the CHBQ in medical students [10, 18]. Currently, there is no instrument like the CHBQ in German-speaking countries that aims to evaluate medical students’ attitude and beliefs towards CM.

The aim was to examine the validity and reliability of the German translation of the CHBQ that we used in our study, assessing medical students’ attitudes and beliefs about CM [18].

## Materials and methods

### Participants and procedure

To examine the validity and reliability of the CHBQ we performed a methodological study nested in a cross-sectional study. The methodological study comprised two phases. In phase 1, a translation and adaptation of the CHBQ from English to German was undertaken. In phase 2, psychometric validation of the CHBQ version was determined. The methodological study used data from first- and fifth-year medical students, enrolled at Charité – Universitätsmedizin Berlin, who participated in an online-exploratory cross-sectional study at the beginning of the summer term 2019 (for further details and on the recruitment procedure, see [18]). All participants were informed about the study purpose and data protection via an online text. Informed consent was provided prior to participation [18]. The study was conducted in accordance with the standards of the Declaration of Helsinki and the International Council for Harmonisation of Technical Requirements for Pharmaceuticals for Human Use (ICH)- good clinical practice (GCP) guidelines, and ethical approval was granted by the Charité ethics committee (EA1/033/19).

### CAM health belief questionnaire (CHBQ)

The CHBQ was developed by Lie and Boker [11] to measure medical students’ attitudes and beliefs to help facilitate further research into CM curriculum development and to systematically measure progress of learning outcomes. The original English version demonstrated acceptable internal consistency in the validation study, with a Cronbach’s alpha of α = 0.75.

The CHBQ consists of 10 items rated on a 7-point scale ranging from 1 (absolutely disagree) to 7 (absolutely agree). All 10 items are summed to form the CHBQ total score, which ranges from 10 to 70 points. A higher score indicates a more positive attitude toward CM. Three of the 10 items (items 6, 7 and 8) are worded negatively and must be reverse coded prior to analysis. For instance, item 7 reads: “Treatments not tested in a scientifically recognized manner should be discouraged”. Assuming that respondents would be more likely to agree with the other items, they would have to disagree with these three items in order to be consistent in their responses. This approach helps to minimize the tendency to answer questions in an affirmative manner.

### Translation and validation process

**Fig. 1:**
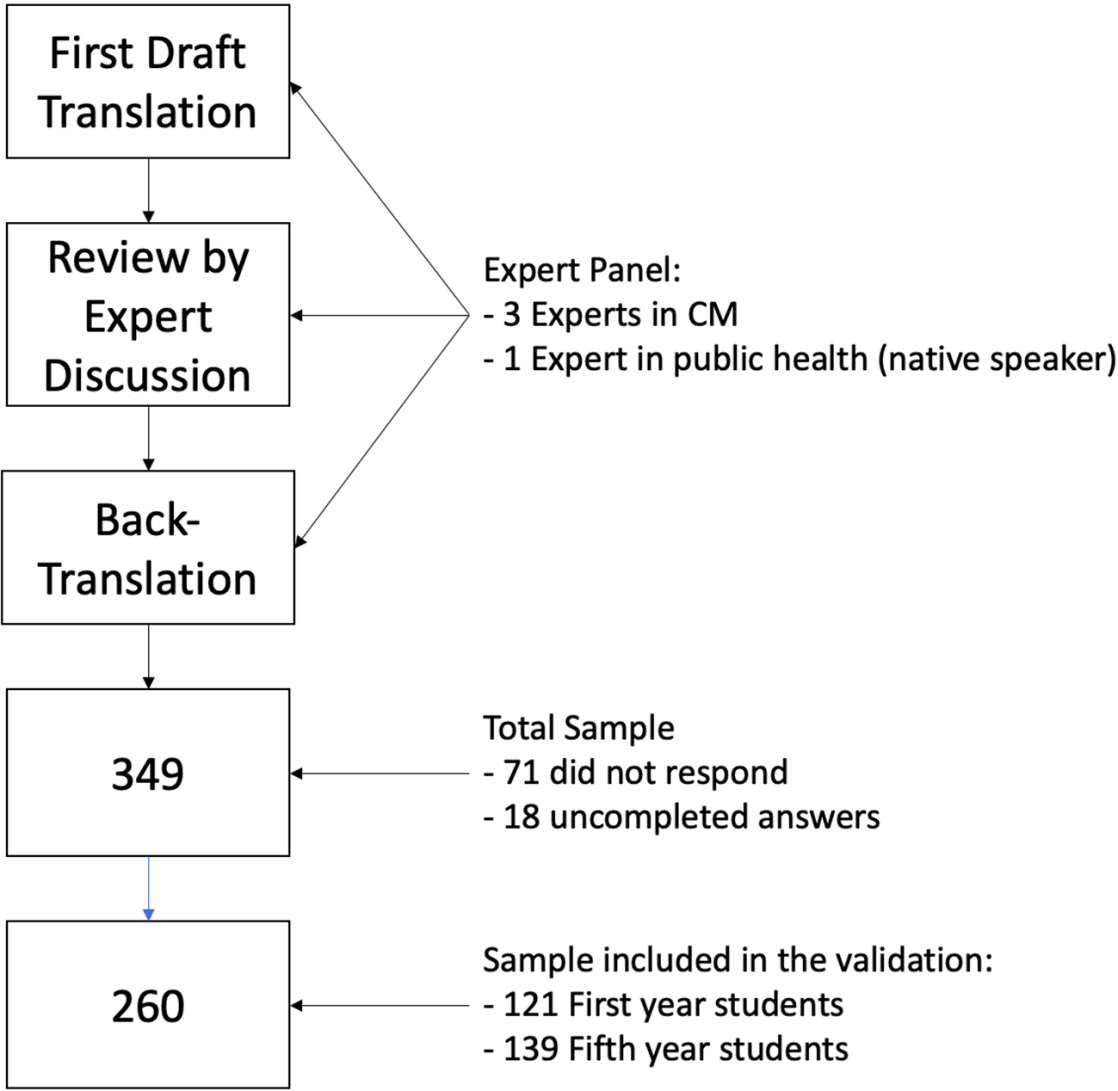
Flow-chart of the study phases: Translation, back-translation and analysis

The German translation of the CHBQ aimed to provide a conceptual equivalence of each item rather than a word for word translation. The CHBQ was translated into German and back-translated into English in accordance with an expert panel consisting of four academic researchers (n = 3 experts in CM, n = 1 expert in public health, see Fig. 1), three of whom were native German speakers and one native English speaker (all experts had a very good command of the respective language in addition to their native language). Firstly, the original English version of the CHBQ was translated into German by each of the three native German-speaking experts to ensure that content, concepts and discrepancies between the original English version and the translated German version were adequately captured. Secondly, the individual translations were reviewed and combined into a first draft questionnaire via discussion by the expert panel in a working group meeting. This draft was then back-translated into English by the four-person expert panel (to check for conceptual equivalence). Lastly, the expert panel agreed on the final translation, and the German version was approved by the senior author. Instructions for the CHBQ respondents remained the same as in the English original: “Please read and respond to each of the 10 statements below by (choosing) the number that most agrees with your beliefs” [11].

### Statistical analysis

Data were analyzed descriptively using mean values of all CHBQ items and standard deviation for continuous variables. Statistical analyses were performed in R (version 4.0.0) [19] and RStudio (version 1.2.5042) [20] using the following packages: *tidyverse* [21, 22], *ggplot2* [21], the *easystats* ecosystem [23–32], *psych* [33], *lavaan* [34, 35] and *gtsummary* [36].

### Construct validity

An exploratory factor analysis (EFA) was performed to determine the factor solution of our German CHBQ translation. After checking the prerequisites for performing an EFA (Kaiser-Meyer-Olkin (KMO), Bartlett’s test for sphericity), the EFA was performed using the R packages *psych* [33], *sjplot* [26], *sjmisc* [25], taking into account the recommendations of Field [37, 38] and Revelle [39] using Ordinary Least Squares (OLS) to determine the minimum residual (minres) solution. Owing to the lack of consensus on the most appropriate method for determining the ideal number of factors, we used the technique implemented in the R package *psycho* by Makowski [40], within the *parameters* package [41]. This approach uses 19 different methods to determine the best consensus between methods to estimate the number of factors to be extracted. Oblimin rotation was tested, but findings indicated no benefit. Therefore no rotation was used for factor extraction.

### Internal consistency

Internal consistency of the CHBQ scale was analyzed using the R packages *psych* [33, 42], *sjstats* [27], *sjPlot* [26], *performance* [29] and *report* [32]. Psychometric assessment was performed by analyzing mean, skewness, kurtosis, item difficulty, item discrimination, and Cronbach’s alpha [43] for each item if it were to be deleted and for the entire scale itself. All measures were interpreted in accordance with the recommendations made by Field [37, 38], Kline [44] and Zinbarg et al. [42]. Revelle and Condon [39] suggest that at least three reliability measures should be reported and interpreted. Therefore we used the *reliability* function in the R package *psych* [33] which incorporates Cronbach’s alpha, McDonald’s omega as an estimate of overall factor saturation and split-half reliability by sampling of multiple combinations of item splits. Moreover, the recommendations suggest that Split-half reliability should be specified as the lowest and highest calculated variant.

## Results

### Sample characteristics

The total sample included 349 medical students. Of these, 278 students returned the part containing the CHBQ, and 260 students completed the CHBQ questionnaire in full (see Fig. 1). One hundred and twenty-one were first- and second-semester students, 139 were ninth- and tenth-semester students. The average age was 23.7 years (± 4.3 SD). The sample included 180 females, 79 males and one did not specify their gender (see Table 1).

**Table 1:** Participant characteristics by semester group and overall sample included in the validation study

Semester	Fifth year (N = 121)	Fifth year (N = 139)	Overall (N = 260)
**Age**			
Mean (SD)	21.6 (4.0)	25.6 (3.7)	23.7 (4.3)
Median [min, max]	20.0 [18.0, 38.0]	24.0 [22.0, 40.0]	23.0 [18.0, 40.0]
**Gender**			
Female	85 (70.2%)	95 (68.3%)	180 (69.2%)
Male	35 (28.9%)	44 (31.7%)	79 (30.4%)
Not specified	1 (0.8%)	0	1 (0.4%)

N, number; SD, standard deviation; min, minimum; max, maximum

### CHBQ scale

Descriptive statistics for all scale items and reliability data are shown in Table 3. Mean scores for the individual items ranged from 3.50 to 5.64 on the 7-point scale (1 = absolutely disagree, 7 = absolutely agree). The complete CHBQ scale had a mean value of M = 44.34 (± 10.44). All items had a range of 7 (1 to 7), with some items being more skewed (items 5, 6 and 9, see Table 1) than others (items 2, 3, 4 and 8). The item with the lowest mean score was item 7: “Treatments not tested in a scientifically recognized manner should be discouraged” (M = 3.50), whereas item 5 had the highest agreement: “A patient’s expectations, health beliefs and values should be integrated into the patient care process” (M = 5.64).

### Construct validity

An exploratory factor analysis was carried out to determine the construct validity of the German translation.

The Kaiser-Meyer-Olkin (KMO) measure of sampling adequacy suggested that the data were appropriate for factor analysis (KMO = 0.87). Bartlett’s test of sphericity also indicated significant correlation in the data for factor analysis (Chisq (45) = 1085.69, p < 0.001).

The results from the factor estimation indicated that 6 of the 19 (31.58%) methods supported a single factor solution (Bentler, Acceleration factor, Scree (SE), Scree (R2), VSS complexity 1, Velicer’s MAP). Other methods estimated between 2 and 7 factors. The method used here is based on maximum consensus and one factor solution had the most consensus.

All ten items within the unidimensional one latent factor solution (with no rotation used) had factor loadings between 0.45 and 0.79 and accounted for 39.60% of the total variance (eigenvalue 3.96). The use of a rotation method (oblimin) had no benefit in explaining the variance and a rotation method was not used in relation to the content design of the scale with a single-factorial solution. Consequently, all ten items were retained for further reliability analysis. EFA results for all ten items with factor loadings are shown in Table 2.

**Table 2:** Exploratory factor analysis - Factor loadings for all ten items of the CAM health belief questionnaire - German version

Variable	Factor 1	Uniqueness
CHBQ_1	0.63	0.60
CHBQ_2	0.65	0.58
CHBQ_3	0.55	0.70
CHBQ_4	0.64	0.60
CHBQ_5	0.45	0.80
CHBQ_6	0.67	0.55
CHBQ_7	0.56	0.68
CHBQ_8	0.59	0.65
CHBQ_9	0.70	0.50
CHBQ_10	0.79	0.38

### Internal consistency

Results of the internal consistency reliability analysis are shown in Table 3. Cronbach’s alpha for the single latent factor structure was 0.86, indicating good reliability [37]. The mean inter-item-correlation revealed an acceptable correlation of 0.39, and the median correlation was similar (0.38). Omega_h was 0.70 and omega total was also good with 0.88 [33, 39, 42]. Item difficulties ranged between 0.50 and 0.81 and can be considered good [33, 39, 42]. Split half reliability was very good with a maximum value of 0.91 (lambda 4) and a minimum value of 0.78 (beta) [33, 39, 42].

**Table 3:** CAM Health belief questionnaire (CHBQ) – Original English item, German translation, item characteristics and reliability measures

	Items - Original English version	Items - German translation	Mean	SD		Skew	Kurtosis	Item difficulty	Item discrimination		Item correlation	α if deleted	
1	The physical and mental health are maintained by an underlying energy or vital force.	Die körperliche und geistige Gesundheit wird durch eine grundlegende Energie oder eine Lebenskraft aufrechterhalten.	4.25	1.78		-0.34	-1.00	0.61	0.59		0.38	0.85	
2	Health and disease are a reflection of balance between positive life-enhancing forces and negative destructive forces.	Gesundheit und Krankheit sind ein Ausdruck des Gleichgewichts zwischen positiven lebensfördernden Kräften und negativen destruktiven Kräften.	3.63	1.63		-0.02	-0.90	0.52	0.61		0.38	0.85	
3	The body is essentially self-healing and the task of a health care provider is to assist in the healing process.	Der Körper ist im Wesentlichen selbstheilend und die Aufgabe eines Gesundheitsversorgers ist es, den Heilungsprozess zu unterstützen.	4.22	1.56		-0.12	-0.69	0.60	0.50		0.40	0.85	
4	A patient’s symptoms should be regarded as a manifestation of a general imbalance or dysfunction affecting the whole body.	Die Symptome eines Patienten sollten als Ausdruck eines allgemeinen Ungleichgewichts oder einer Dysfunktion angesehen werden, die den ganzen Körper betrifft.	4.24	1.58		-0.23	-0.67	0.61	0.59		0.38	0.85	
5	A patient’s expectations, health beliefs and values should be integrated into the patient care process.	Die Erwartungen, gesundheitlichen Ansichten und Werte eines Patienten sollten in den Prozess der Patientenversorgung integriert werden.	5.64	1.28		-1.18	1.55	0.81	0.40		0.41	0.86	
6	Complementary therapies are a threat to public health.*	Komplementäre Therapien sind eine Bedrohung für die öffentliche Gesundheit.*	5.33	1.43		-0.74	0.02	0.76	0.62		0.38	0.84	
7	Treatments not tested in a scientifically recognized manner should be discouraged.*	Behandlungen, die nicht auf wissenschaftlich anerkannte Weise getestet wurden, sollten nicht empfohlen werden.*	3.50	1.75		0.29	-0.90	0.50	0.53		0.39	0.85	
8	Effects of complementary therapies are usually the result of a placebo effect.*	Die Wirkung komplementärer Therapien ist in der Regel das Ergebnis eines Placebo-Effekts.*	4.01	1.65		-0.04	-0.86	0.57	0.55		0.39	0.85	
9	Complementary therapies include ideas and methods from which conventional medicine could benefit.	Zu den komplementären Therapien zählen Ideen und Methoden, von denen die Schulmedizin profitieren könnte.	5.15	1.39		-0.58	-0.03	0.74	0.64		0.37	0.84	
10	Most complementary therapies stimulate the body’s natural therapeutic powers.	Die meisten komplementären Therapien stimulieren die natürlichen therapeutischen Kräfte des Körpers.	4.38	1.53		-0.34	-0.32	0.63	0.72		0.36	0.84	

*Items were reverse coded for analysis and interpretation. N = 260. Mean inter item correlation = 0.387. Cronbach’s alpha for scale: 0.862

## Discussion

The German translation of the CHBQ, presented here for the first time, showed to be a reliable instrument (α = 0.86) with a single factor solution for measuring health attitudes and beliefs towards CM among medical students in Germany.

By using a reliable and validated German-language instrument to measure attitudes of health beliefs towards CM, there is the potential for a broader application for quality assessment and further development of CM education in Germany.

Overall, our findings are comparable to those reported by Lie & Boker [11] for the original English version of the CHBQ. According to Lie & Boker, the individual item mean scores ranked between M = 4.1 and M = 5.9 on the 7-point scale with an overall mean scale score of M = 47.8. In addition, our study showed comparable scores ranging from M = 3.5 to M = 5.64, with an overall mean score of M = 44.3. Interestingly, in both our study and that of Lie & Boker, the same statement had the lowest agreements: *“Treatments not tested in a scientifically recognized manner should be discouraged”* (CHBQ item 7) and *“A patient’s expectations, health beliefs and values should be integrated into the patient care process”* (CHBQ item 5) had the highest agreement. It is not clear why these two statements receive particularly low or high levels of agreement, but it could be because these statements contain statements that may be general norms or shared values by an American and German society and are widely held.

Results of the EFA revealed a unidimensional factor loading. Only Item 5, *“A patient’s expectations, health beliefs and values should be integrated into the patient care process”*, showed a weak factor loading (0.45, see Table 2). Interestingly, it is precisely this item number 5, that had the highest agreement among all participants. Item 5 also demonstrated the highest uniqueness of all items in the scale (0.80, see Table 3), as well as a high item difficulty (0.81) and low item discrimination (0.40). Therefore, this statement could potentially be excluded from the scale, as the reliability of the overall scale would not change as a result (α if item deleted = 0.86). Nevertheless, owing to its practical relevance, we decided to retain the item in the scale.

To date, the CHBQ has been used in numerous studies [11, 14–18, 45–47], but few have performed psychometric validation of the scale, especially when translated into other languages. In addition to the original version of the CHBQ, who performed psychometric analysis, a version was used on Czech pharmacy students [16]. In this study, the mean score of the CHBQ was 48.5. There was a tendency of agreement towards CM, too, similar to our study. The mean score was above the midpoint of 40. A factor analytic review of the structure of the scale and a psychometric evaluation was not performed in the Czech version.

Another translation of the CHBQ was performed in two studies by Samuels et al. [46, 47] in Israel. In the first study, data from 173 nurse-midwives in 5 study centers were analyzed. In this study, an exploratory factor analysis was performed as well, and a three-factor solution with 62% variance was extracted for the CHBQ scale. Cronbach’s alpha was = 0.81 for the entire scale. In another study of 170 obstetricians during pregnancy and childbirth [46], the version previously translated into Hebrew was used again. In this study, a three-factor solution was also extracted using factor analysis with 63.1% variance resolution, and the reliability of the total scale with Cronbach’s alpha was = 0.82. The mean score of the CHBQ scale in this study was 40.4 points, slightly above the midpoint, with a slight tendency toward agreement with CM. Also in these two studies, the items with patient-centered statements, especially item 5 on integrating patient opinions and health beliefs into the care process, were the items with very strong agreement.

In our study, we have confirmed very good reliability of the German version of the CHBQ using Cronbach’s alpha and split half reliability. The Cronbach’s alpha test value for the whole scale was α = 0.86, which is slightly better than the original English version (Cronbach’s α = 0.75). Given that, we found only a one factor solution for the CHBQ scale and Cronbach’s alpha values for the individual items were also very good, it did not seem sensible to remove individual items from the scale. Compared to the other studies from Israel [46, 47] and the original study [13], our German translation has comparable and slightly improved reliability. In future studies, the construct validity of the scale should be further determined using confirmatory factor analysis (CFA) with a larger sample size to confirm the factor structure of the scale. Especially since the Israeli studies [46, 47] found a three factor solution and in the English original and in our German translation only a one factor solution was used. In the present study, the sample size was too small to further investigate the latent structure via CFA.

While the German CHBQ version has so far only been used and validated to assess students’ beliefs and attitudes, the questionnaire could also be used in other healthcare settings. For instance, not only for the purpose of quality assessment in health education, but also to determine patients’ views and health beliefs of other professions in health care settings. Like in the Israeli setting, the scale was used with already working professionals. Therefore, the scale should be validated in other populations, e.g. different patient groups, with physicians or nurses, to verify its usefulness for quality assurance by capturing patients’ attitudes and expectations within health care settings.

### Limitations

Our study used a similar study population to the original English validation study [11]. While this enables a comparison with the original study, a sample consisting of students from a single university and from only two cohorts (four semesters) represents a limited population. Thus, the results cannot be generalized to other groups of individuals.

Due to the cross-sectional nature of the study, no change over time could be assessed and therefore, no conclusions can be drawn about the sensitivity to changes of medical students’ attitude towards CM along their medical education. Furthermore, the sample was not recruited specifically for the purpose of validating the CHBQ. In addition, we performed no pilot testing of the translated version on a small sample prior to using the scale in the original study [18], which would have been desirable for optimizing the translation and validation process.

## Conclusion

Our study results indicate that the German translation of the CHBQ is a reliable and valid scale to assess students’ health beliefs and attitudes towards CM.

## Data Availability

The datasets generated and analyzed during the current study are not publicly available due to the participants not having given consent for data sharing and due to concerns that the individual privacy of the participants could be compromised, but are available from the corresponding author on reasonable request.

## Abbreviations

CAM: Complementary and Alternative Medicine
CHBQ: CAM Health Belief Questionnaire
CM: Complementary Medicine
EFA: Exploratory Factor Analysis
IMAQ: Integrative Medicine Attitude Questionnaire

## References

[1] Ernst E (2008). [Complementary medicine - a critical analysis]. Wien Med Wochenschr.

[2] NCCIH. National Center for Complementary and Integrative Health. 2023 Available from: https://www.nccih.nih.gov/.

[3] WHO. Benchmarks for training in traditional / complementary and alternative medicine: benchmarks for training in naturopathy 2010.

[4] Clearinghouse N. Complementary, Alternative, or Integrative Health: What’s In a Name? 2021.

[5] Cramer H (2021). We’re still the Blue Journal-Introducing Journal of Integrative and complementary medicine. J Altern Complement Med.

[6] Esch T, Brinkhaus B (2020). Neue Definitionen Der Integrativen Medizin: Alter Wein in Neuen Schlauchen?. Complement Med Res.

[7] Harris PE, Cooper KL, Relton C, Thomas KJ (2012). Prevalence of complementary and alternative medicine (CAM) use by the general population: a systematic review and update. Int J Clin Pract.

[8] Thanner M, Nagel E, Loss J (2014). [Complementary and alternative medicine in the German outpatient setting: extent, structure and reasons for provision]. Gesundheitswesen.

[9] Linde K, Alscher A, Friedrichs C, Wagenpfeil S, Karsch-Volk M, Schneider A (2015). Belief in and use of complementary therapies among family physicians, internists and orthopaedists in Germany - cross-sectional survey. Fam Pract.

[10] Quartey NK, Ma PH, Chung VC, Griffiths SM (2012). Complementary and alternative medicine education for medical profession: systematic review. Evid Based Complement Alternat Med.

[11] Lie D, Boker J (2004). Development and validation of the CAM Health Belief Questionnaire (CHBQ) and CAM use and attitudes amongst medical students. BMC Med Educ.

[12] Schneider CD, Meek PM, Bell IR (2003). Development and validation of IMAQ: Integrative Medicine attitude questionnaire. BMC Med Educ.

[13] Lie D, Boker J (2004). Development and validation of the CAM Health Belief Questionnaire (CHBQ) and CAM use and attitudes amongst medical students. BMC Med Educ.

[14] Xie H, Sang T, Li W, Li L, Gao Y, Qiu W, Zhou H (2020). A survey on perceptions of complementary and alternative medicine among undergraduates in China. Evid Based Complement Alternat Med.

[15] Walker BF, Armson A, Hodgetts C, Jacques A, Chin FE, Kow G (2017). Knowledge, attitude, influences and use of complementary and alternative medicine (CAM) among chiropractic and nursing students. Chiropr Man Therap.

[16] Pokladnikova J, Lie D (2008). Comparison of attitudes, beliefs, and resource-seeking behavior for CAM among first- and third-year Czech pharmacy students. Am J Pharm Educ.

[17] Jakovljevic MB, Djordjevic V, Markovic V, Milovanovic O, Rancic NK, Cupara SM (2013). Cross-sectional survey on complementary and alternative medicine awareness among health care professionals and students using CHBQ questionnaire in a Balkan country. Chin J Integr Med.

[18] Rotter G, Jerzynski L, Hinse M, Binting S, Brinkhaus B (2021). The attitude of medical students toward complementary medicine: results of a cross-sectional study. J Altern Complement Med.

[19] Core Team R. R: a language and environment for statistical computing. Vienna, Austria: R Foundation for Statistical Computing; 2020 Available from: https://www.R-project.org/.

[20] RStudio Team. RStudio: Integrated Development for R, RStudio. Inc., Boston, MA 2020 Available from: http://www.rstudio.com/.

[21] Wickham H (2016). ggplot2: elegant graphics for data analysis.

[22] Wickham H, Averick M, Bryan J, Chang W, McGowan L, François R (2019). Welcome to the Tidyverse. J Open Source Softw.

[23] Lüdecke D. ggeffects: Create tidy data frames of marginal effects for ‘ggplot’ from model outputs. 2021.

[24] Lüdecke D. sjlabelled: Labelled data utility functions. manual. 2021.

[25] Lüdecke D. sjmisc: Data and variable transformation functions. manual. 2021.

[26] Lüdecke D. sjPlot: Data visualization for statistics in social science. 2021.

[27] Lüdecke D. sjstats: Collection of convenient functions for common statistical computations. manual. 2021.

[28] Lüdecke D. strengejacke: Load packages associated with strenge jacke! manual. 2019.

[29] Lüdecke D, Ben-Shachar M, Patil I, Waggoner P, Makowski D (2021). Performance: an R Package for Assessment, comparison and testing of statistical models. J Open Source Softw.

[30] Lüdecke D, Waggoner P, Makowski D (2019). Insight: a unified interface to Access Information from Model objects in R. J Open Source Softw.

[31] Makowski D, Ben-Shachar M, Patil I, Lüdecke D (2020). Methods and Algorithms for Correlation Analysis in R. J Open Source Softw.

[32] Makowski D, Lüdecke D, Ben-Shachar MS. Patil I. report: Automated reporting of results and statistical models. 2021.

[33] Revelle W. psych: Procedures for psychological, psychometric, and personality research. 2021.

[34] Kline RB. Principles and practice of structural equation modeling. Fourth edition. ed. New York: The Guilford Press; 2016. xvii, 534 pages p.

[35] Rosseel Y (2012). Lavaan: an R Package for Structural equation modeling. J Stat Softw.

[36] Sjoberg DD, Curry M, Hannum M, Larmarange J, Whiting K, Zabor EC. gtsummary: Presentation-ready data summary and analytic result tables. manual. 2021 2021.

[37] Field AP, Miles J, Field Z. Discovering statistics using R. London: SAGE Publications, Inc.; 2012 2012.

[38] Field AP, Wilcox RR (2017). Robust statistical methods: a primer for clinical psychology and experimental psychopathology researchers. Behav Res Ther.

[39] Revelle W, Condon DM (2019). Reliability from alpha to omega: a tutorial. Psychol Assess.

[40] Makowski D. The Psycho Package: an efficient and publishing-oriented workflow for Psychological Science. JOSS. 2018;3(22).

[41] Lüdecke D, Ben-Shachar M, Patil I, Makowski D, Extracting (2020). Computing and exploring the parameters of statistical models using R. J Open Source Softw.

[42] Zinbarg RE, Revelle W, Yovel I, Li W (2005). Cronbach’s α, Revelle’s β, and Mcdonald’s ωH: their relations with each other and two alternative conceptualizations of reliability. Psychometrika.

[43] Cronbach LJ (1951). Coefficient alpha and the Internal structure of tests. Psychometrika.

[44] Kline RB. Beyond significance testing: Statistics reform in the behavioral sciences (2nd ed.). Washington: American Psychological Association; 2013 2013.

[45] Riccard CP, Skelton M (2008). Comparative analysis of 1st, 2nd, and 4th year MD students’ attitudes toward complementary Alternative Medicine (CAM). BMC Res Notes.

[46] Samuels N, Zisk-Rony RY, Many A, Ben-Shitrit G, Erez O, Mankuta D (2013). Use of and attitudes toward complementary and alternative medicine among obstetricians in Israel. Int J Gynecol Obstet.

[47] Samuels N, Zisk-Rony RY, Singer SR, Dulitzky M, Mankuta D, Shuval JT, Oberbaum M (2010). Use of and attitudes toward complementary and alternative medicine among nurse-midwives in Israel. Am J Obstet Gynecol.

